# Supplementary material for: Development of Efficient Photocatalyst MIL-68(Ga)_NH2 Metal-Organic Framework for the Removal of Cr(VI) and Cr(VI)/RhB from Wastewater under Visible Light
Source: Materials (Basel). 2022 May 24;15(11):3761. doi: 10.3390/ma15113761 (PMC9181230; doi:10.3390/ma15113761)
Supplement: Supplementary file 1 [file materials-15-03761-s001.zip › materials-1708514-supplementary.pdf]

Supporting information for

Fabrication of efficient photocatalyst MIL-68(Ga)_NH_2 metal-organic framework for the remove of Cr(VI) and Cr(VI)/RhB from wastewater under visible light

Lei Wu*,[1] Doudou Qin,[1] Fan Fang,[*2] Weifeng Wang[1], Wenying Zhao[1]

[1.] *Institute of Polymer Materials, School of materials Science and Engineering, Chang'an University, Xi'an 710064, China.*

[2.] *Xi'an Thermal Power Research Institute CO.,LTD.*

[*] Corresponding author (email: wulei@chd.edu.cn)

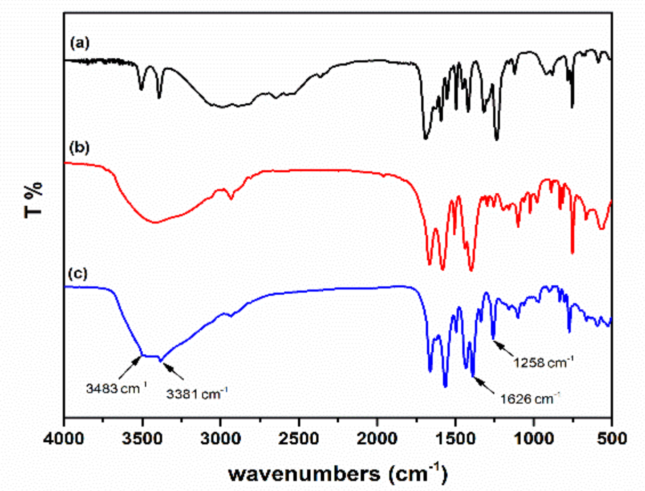

Figure S1. The IR spectra of (a) $H_2BDC-NH_2$; (b) as-synthesized MIL-68(Ga); (c) as-synthesized MIL-68(Ga)_NH_2 samples

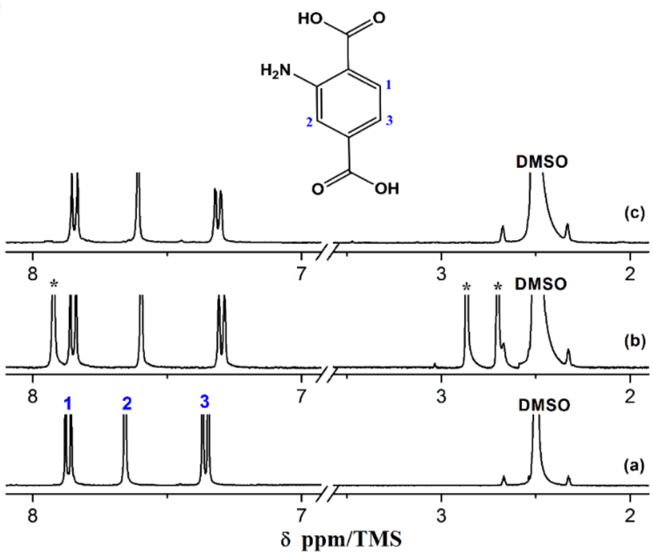

Figure S2. ^1H NMR spectra of (a) $H_2BDC-NH_2$ (b) as-synthesized MIL-68(Ga)_NH_2 (c) activated MIL-68(Ga)_NH_2; * marks denote positions of resonances assignable to DMF protons

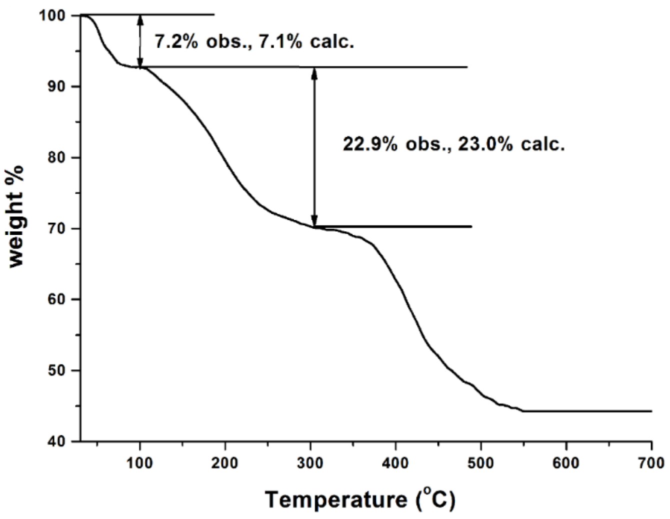

Figure S3. TGA curves of as-synthesized MIL-68(Ga)_NH2 under N_2 flow.

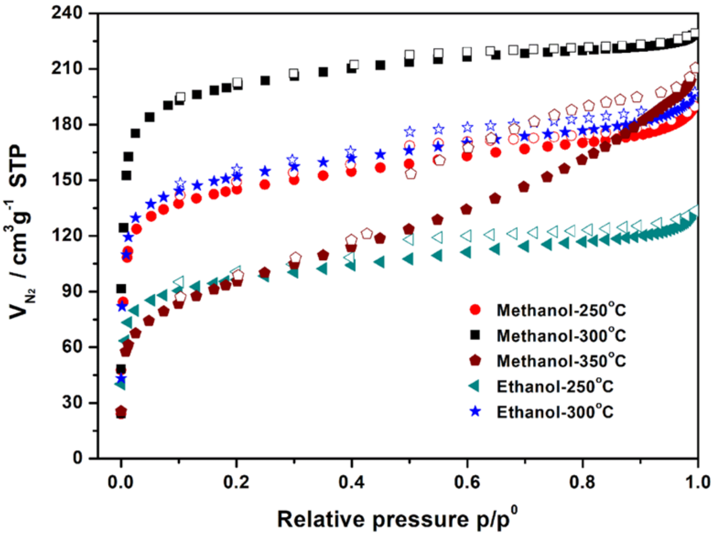

Figure S4. N_2 sorption isotherms of MIL-68(Ga)_NH2 activated at different temperatures. Solid and

open symbols refer to adsorption and desorption branches, respectively.

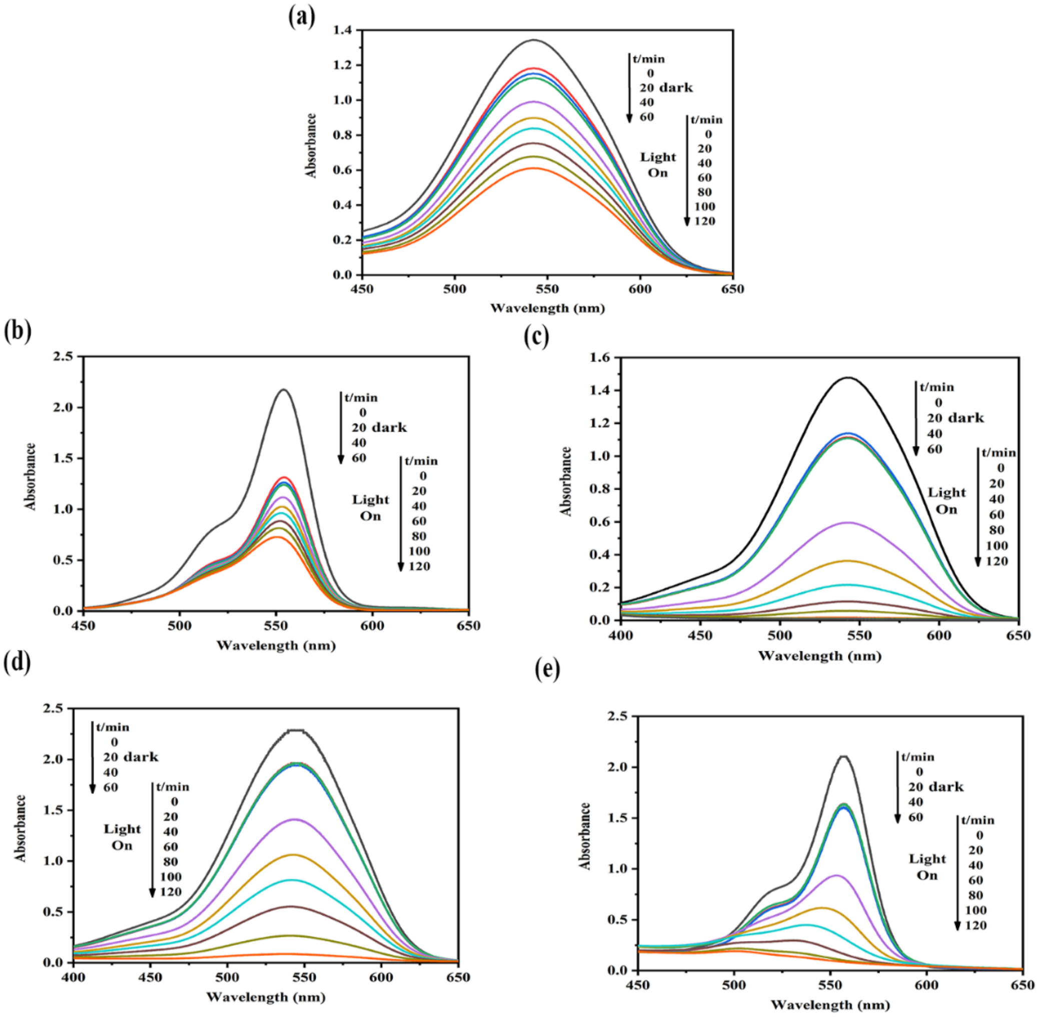

Figure S5. (a) Under Cr(VI) pollutant system, absorbance curve of Cr(VI) over time; (b) Under RhB pollutant system, absorbance curve of RhB over time; (c) Under Cr(VI)/ethanol pollutant system, absorbance curve of Cr(VI) over time; (d) Under Cr(VI)/RhB pollutant system, absorbance curve of Cr(VI) over time; (e) Under Cr(VI)/RhB pollutant system, absorbance curve of RhB over time.

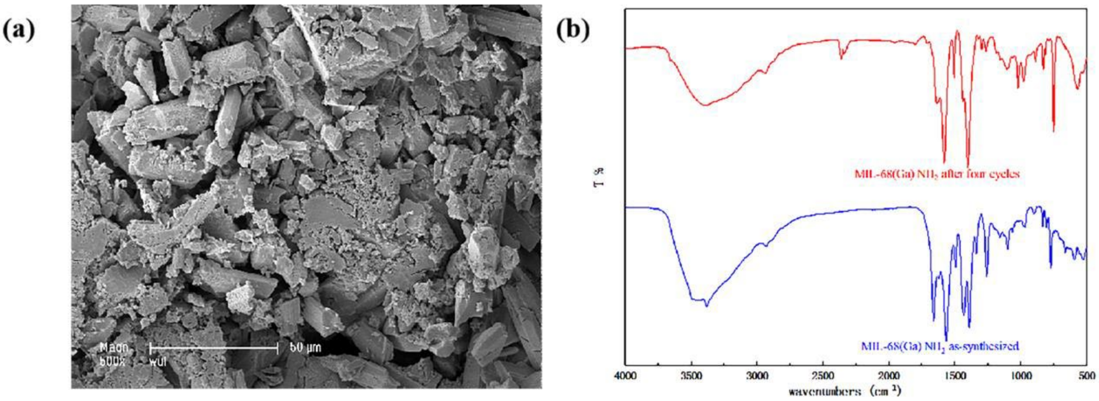

Figure S6. (a) SEM and (b) IR spectra of MIL-68(Ga)_NH_2 after four cycles of photocatalytic degradation experiments.

It can be seen from SEM of MIL-68(Ga)_NH_2 after four cycles of photocatalytic degradation experiments that the morphology of the sample has been damaged after recycling. And the phenomenon of agglomeration and pitting appeared on the appearance of the crystal. The IR spectrum indicates that the skeleton of MIL-68(Ga)_NH_2 has been maintained after cycling. However, the two sharp small peaks assignable to amino group around 3300~3500 cm^{-1} are not obvious due to the coverage of the board peak of water. It may be caused by the adsorption of a large amount of water in the pores during the photodegradation experiment.

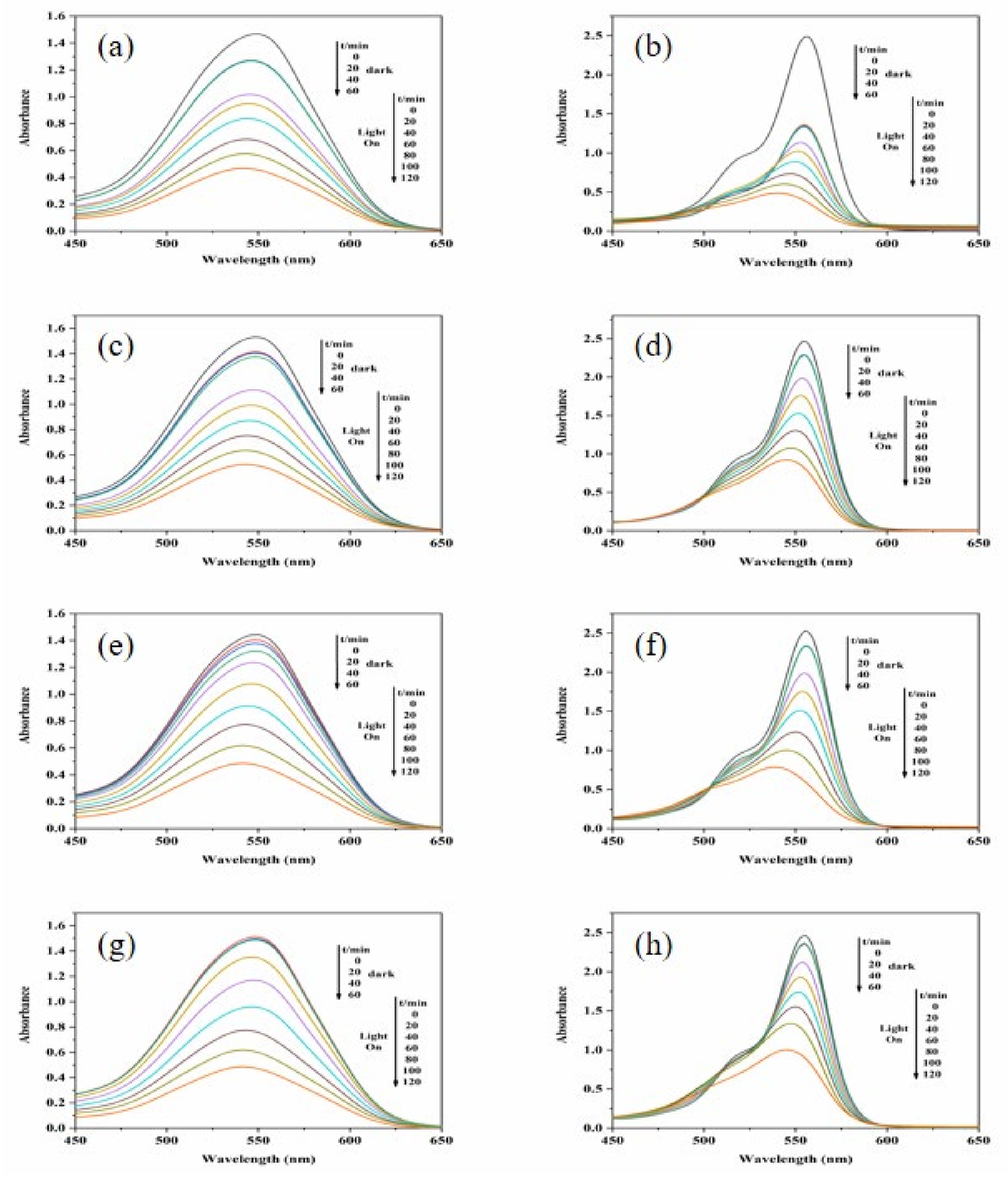

Figure S7. Under Cr(VI)/RhB pollutant system, absorbance curve of (a),(c),(e),(g) Cr(VI) and (b),(d),(f),(h) RhB over time for four times.

(a)

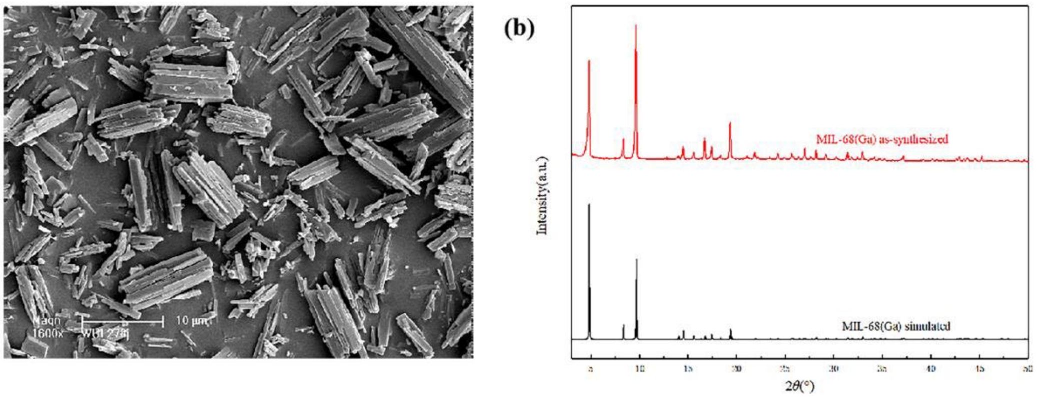

Figure S8. (a) SEM of MIL-68(Ga) and (b) PXRD spectra of as-synthesized MIL-68(Ga) sample and simulated PXRD spectra of MIL-68(Ga).
